# The Importance of Being Dead: Cell Death Mechanisms Assessment in Anti-Sarcoma Therapy

**DOI:** 10.3389/fonc.2015.00082

**Published:** 2015-04-07

**Authors:** Santiago Rello-Varona, David Herrero-Martín, Laura Lagares-Tena, Roser López-Alemany, Núria Mulet-Margalef, Juan Huertas-Martínez, Silvia Garcia-Monclús, Xavier García del Muro, Cristina Muñoz-Pinedo, Oscar Martínez Tirado

**Affiliations:** ^1^Sarcoma Research Group, Molecular Oncology Laboratory, Bellvitge Biomedical Research Institute (IDIBELL), L’Hospitalet de Llobregat, Barcelona, Spain; ^2^Cell Death Regulation Group, Molecular Oncology Laboratory, Bellvitge Biomedical Research Institute (IDIBELL), L’Hospitalet de Llobregat, Barcelona, Spain

**Keywords:** cell death mechanisms, sarcoma, translocation-bearing sarcomas, apoptosis, necrosis, autophagic cell death, mitotic catastrophe

## Abstract

Cell death can occur through different mechanisms, defined by their nature and physiological implications. Correct assessment of cell death is crucial for cancer therapy success. Sarcomas are a large and diverse group of neoplasias from mesenchymal origin. Among cell death types, apoptosis is by far the most studied in sarcomas. Albeit very promising in other fields, regulated necrosis and other cell death circumstances (as so-called “autophagic cell death” or “mitotic catastrophe”) have not been yet properly addressed in sarcomas. Cell death is usually quantified in sarcomas by unspecific assays and in most cases the precise sequence of events remains poorly characterized. In this review, our main objective is to put into context the most recent sarcoma cell death findings in the more general landscape of different cell death modalities.

## Introduction

### Facts

- Sarcomas are a highly heterogeneous group of mesenchymal tumors.
- Among cell death mechanisms, only apoptosis has been extensively studied in sarcomas.
- Fusion proteins, actors of translocation-derived sarcomagenesis, play an anti-apoptotic role in sarcomas.
- Proper and deeper assessment of cell death in sarcomas is mandatory.

### Challenges

- Can we improve the current therapeutic protocols in sarcomas through a better knowledge of cell death mechanisms?
- Can we assess more accurately the sequence of events of every type of cell death?
- Which are the key molecules that determine tumor cell death after therapy?
- Do translocation-bearing sarcomas have specific weaknesses in their cell death signaling networks?

Cancer therapies are aimed to induce the specific destruction of tumor cells without compromising patient health. This makes cell death mechanisms a central point of any therapeutic approach (1, 2). However, no every death is equally desirable in terms of therapy (3). The need of theoretical arrangement in the field has become evident during the past years. Our knowledge on cell death mechanisms has increased enormously and the available methodology has become more and more sophisticated. Therefore, a clear nomenclature based on reliable markers has been proposed (1, 4). Additionally, the growing number of cell death participants have been organized in clear hierarchic frameworks (5).

Sarcomas are a rare and heterogeneous group (more than 50 different clinical and molecular entities) of malignant tumors with mesenchymal origin. Molecular biology of sarcomas has remained elusive until recently, and a better knowledge remains as an unmet need (6). New drugs against potential targets in tumor cells with a crucial role in their metabolism or pro-survival fitness could improve the prognosis of these patients. Indeed, the relatively high rate of therapeutic failure and tumor relapse demands a better assessment of cell death induction. But scientific efforts in this discipline are historically undermined by the relative low investments and isolated work (7).

**Figure 1:**
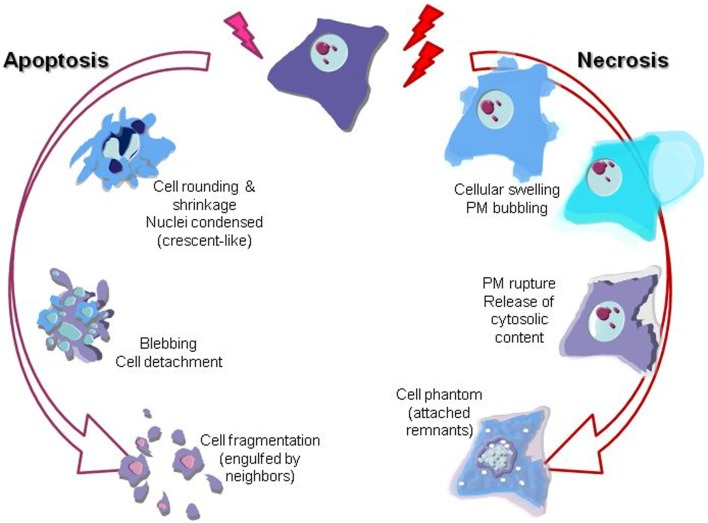
**Graphical illustration of the most prominent morphological features of apoptotic and necrotic cell death mechanisms**. Nuclei changes (karyorrhexis), cytoplasm shrinkage, and blebbing are the most evident descriptors of apoptosis. On the other hand, necrosis is clearly recognizable by cell swelling (loss of osmotic barrier) leading to the plasma membrane (PM) breakage and final release of the inner soluble content and nuclei karyolysis.

**Figure 2:**
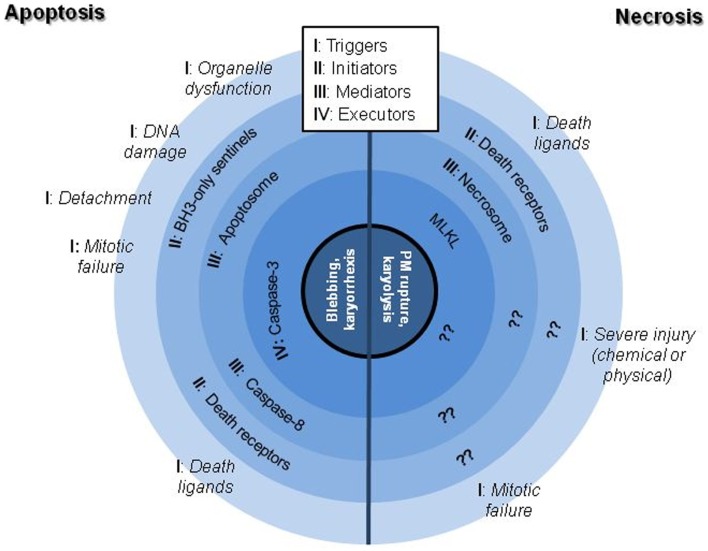
**Schematic representation of the better characterized signaling hubs of apoptotic and necrotic cell death mechanisms**. Note that necrotic processes are substantially worse described than apoptotic ones, being still controversial if the execution phase is protein-driven or result of a massive metabolic failure.

The scientific landscape involving cell death mechanisms in sarcomas can be improved. The majority of articles included in the present review focused on apoptosis (mostly) and necrosis, whose morphological characters (Figure 1) and signaling players (Figure 2) are better described. Many studies about cell death in sarcomas just describe the occurrence of cell death without a proper characterization of the sequence-of-events leading to a particular form of death. The aim of the present review is to help sarcoma researchers to face new knowledge on cell death mechanisms in order to routinely include it in their assessments.

## Cell Death Mechanisms

### Apoptosis

Apoptosis involves a cellular controlled demolition process. Signaling cascades are finely orchestrated and secured, to ensure its perfect onset only when it is required (8). Caspases are the major actors in cellular demolition; once triggered, caspases can cross-activate each other and thus amplify the apoptotic signal (8). Apoptosis is by far the most studied form of cell death in sarcoma research. Nevertheless, researchers either employ uninformative methods about the form of death (i.e., Trypan Blue assay), or the mechanisms leading to such death are not always fully analyzed. Apoptosis recognition is easy by simple morphological features visible under the microscope: nuclear condensation and fragmentation, blebbing etc. (Figure 1). Other techniques (immunofluorescence or western blotting of cleaved caspases and/or caspase substrates, etc.) can be used to monitor specific mediators and executors of the process (9–11). Based on their biochemical features, we can describe two major pathways in apoptotic signaling: the intrinsic or mitochondrial pathway and the death receptor pathway (Figure 2).

#### Mitochondrial apoptosis

The “intrinsic pathway” is defined by the role of the mitochondria as encounter point of most of its initiators and mediators. The Bcl-2 family of proteins controls this pathway by regulating the formation of a pore in the mitochondrial outer membrane (12). Several signaling pathways converge in the regulation of Bcl-2 proteins, from DNA-damage sensor system to organelle stress and malfunction or growth factor signaling (Figure 2) (13, 14). In order to demonstrate that a drug or physiological input induces apoptosis through the mitochondrial pathway, exogenous overexpression of anti-apoptotic Bcl-2 family members can be performed; this should either prevent cell death or switch the mechanism to necrosis.

**Figure 3:**
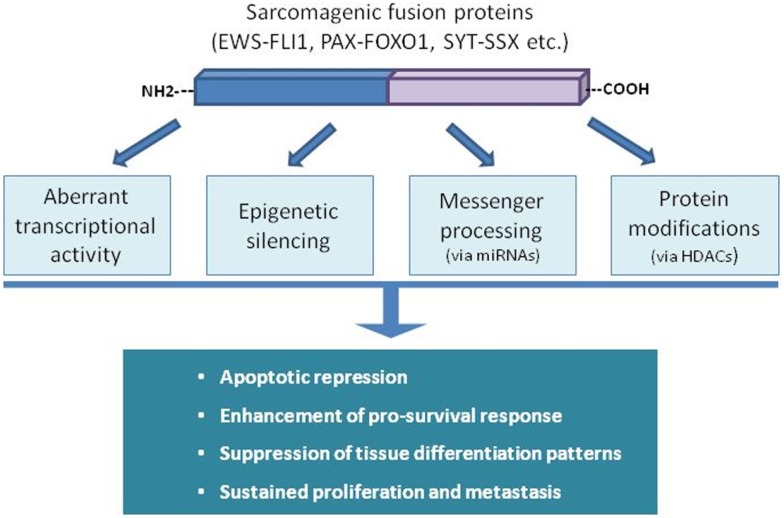
**Fusion proteins in sarcomas disturb the natural physiological balance between pro-survival and death signaling inputs through different ways**. The panoply of mechanisms and cellular targets disturbed demonstrates the powerful tumorigenic effect of a single event of genomic rearrangement.

Some sarcomas rely on the presence of specific aberrant fusion proteins, generated after chromosomal rearrangements. Deregulation of gene expression in sarcomas driven by these chimeric oncoproteins can occur at different levels (epigenetic silencing, transcription activity, messenger processing, etc.) affecting every cellular process, including apoptosis (Figure 3). In the case of Ewing Sarcoma (ES), the fusion proteins EWS-FLI1 or EWS-ERG have an inhibitory effect on part of the apoptotic machinery (15, 16). This effect is mediated by direct or indirect interactions with several signaling pathways modulating apoptosis repression and inducing sustained growth (17–20). Alveolar rhabdomyosarcoma (aRMS) is also dependent on fusion proteins involving different PAX proteins with FOXO1, which also targets different signaling networks in order to ensure evasion of apoptosis (21, 22). SYT-SSX chimera proteins are present in the majority of synovial sarcoma tumors. They are involved in resistance to pro-apoptotic stimuli by modulating the levels and the activity of key apoptotic players of the Bcl-2 family of proteins (23). Furthermore, certain translocation-bearing sarcomas are also characterized by failure to complete tissue differentiation (i.e., RMS to skeletal muscle, liposarcoma to adipocytes) in a process mediated by their specific fusion protein and linked to the inhibition of apoptosis induction (24–26). Several recent studies have linked miRNAs status with apoptosis regulation in chromosome translocation-bearing sarcomas. Hence, mitochondrial apoptotic resistance in ES correlates with miR-125b upregulation through p53 and Bak (27) but overexpression of miR-206 in RMS promotes proliferation arrest and some sort of cell death (28). Overexpression of miR-145 and miR-451 in liposarcoma cell lines decreases cellular proliferation, impairs cell cycle progression, and boosts cell death (29), whereas overexpression of miR-26a-2 has the opposite effects (30).

The levels and status of key pro- and anti-apoptotic proteins are also crucial for understanding the differential sensitivity of cells toward apoptosis. Most ES cells have both the p53 downstream pathway and the DNA-damage signaling pathway functionally intact. The resistance of some ES cell lines to p53-induced apoptosis has been linked to a high Bcl-2/Bax ratio and low levels of Apaf-1 (31). However, the influence of fusion proteins inactivates p53 by deacetylation at Lys-382 driven by both EWS-FLI1 and HDAC1 (32), meaning that re-expression or re-activation of p53 could be a good strategy against these tumors. Similar phenomena occur in other fusion-positive sarcomas and accordingly, histone deacetylase inhibitors have been successfully tested as apoptotic inducers in different sarcoma types (33, 34). p53 re-activator agents as Nutlin-3 and/or PRIMA-1 are able to induce apoptosis successfully through Noxa, Puma, or p21 upregulation in both mutant and wild-type p53 sarcoma cell lines (35–37). Among downstream p53 targets p21, c-Myc, Bax, MDM2, DRAL, Bcl-2, and Bcl-x_L_ have been suggested as key apoptotic regulators in different sarcoma models (38–43). Plasma membrane-anchored growth receptors such as NGFR or IGF-1R have an anti-apoptotic role (44, 45). In contrast, distinct behaviors have been suggested for the closely related receptors PDGFR α and β (46). Thus, PDGFR α favors cellular stemness and PDGFR β promotes angiogenesis in the tumor stroma. Hepatocyte-growth-factor activator inhibitors (HAI-1 and HAI-2) act as tumor suppressors leading to apoptosis and necrosis in leiomyosarcoma (47). Also, inhibition of endogenous tyrosine kinase B (TrkB) signaling suppresses cell proliferation and increases apoptosis in cultured leiomyosarcoma cells (48). In this context, tyrosine kinase inhibitors like Sorafenib induce apoptosis on many leiomyosarcoma or synovial sarcoma cell lines by inhibiting the RAF/MEK/ERK signaling pathway, among others (49, 50). Apoptotic cascades induced by other kinase inhibitors like JAK1 and 2 have been analyzed in detail in RMS and ES cells. These inhibitors lead to the alteration of the balance between the pro-apoptotic Bax and the anti-apoptotic proteins Bcl-2 and Bcl-x_L_, the release of cytochrome *c*, and the activation of caspase-9, -8, and -3 (51, 52).

Many other different strategies have been used in sarcomas to induce mitochondrial apoptosis. Betulinic acid is able to target the mitochondria in ES, promoting the permeabilization of the outer membrane resulting in the release, from the mitochondria to the cytosol, of soluble factors such as AIF and cytochrome *c*, who ultimately leads to caspase activation (53). Direct targeting of mitochondrial physiology was also explored in RMS with photodynamic therapy (54) and ROS-generation agents (55). Proteasome inhibitors as Bortezomib generate a major stress in the cell machinery, triggering a number of different reactions, many of them aimed to induce apoptosis. Bortezomib has been successfully employed in different pre-clinical models (56, 57). Heat shock proteins are among the most important actors against protein stress in cells. Accordingly, Hsp-90 antagonists had been shown to induce transient growth arrest and apoptosis in RMS cells (58). Likewise, some metabolic disruptors like 2-deoxyglucose, Lovastatin, and Catechins have been successfully tested as promoters of mitochondrial apoptosis by unbalancing the equilibrium of Bcl-2 family of proteins (59–61). Furthermore, down-regulation of inhibitor of apoptosis proteins (IAPs) also leads to apoptosis, identified by PARP cleavage, in pediatric sarcomas (62).

To keep their correct physiology, cells rely in their interaction with neighbors and microenvironment, meaning that detachment is a major apoptotic trigger. The process of detachment-induced apoptosis is termed anoikis (4). The lack of attachment activates signals from the plasma membrane, mostly by integrins and the focal adhesion kinase (FAK) that regulate the BH3-only proteins through the mitochondrial commitment to cell suicide (Figure 2) (63). Cell culture in non-adherent conditions, like soft-agar, is the better way to study this process. Suppression of anoikis cell death is considered an important hallmark of transformed cells and thus, a pre-metastatic key process (64).

Anoikis resistance in sarcomas has been described to be associated with integrins, Bcl-2 and caspase-8, CD99 isoforms, RANK, and ERK (65–68). ES cells survival in non-adherent conditions is mediated by E-cadherin dependent spheroid formation, avoiding apoptotic triggering by means of the PI3K/Akt pathway (69). Scotlandi *et al*. demonstrated the relevance of IGF-1R in the anoikis-resistant ES cell line TC71. Impairment of IGF-1R signaling (by neutralizing antibodies or siRNAs expression) led to a lower survival in anchorage-independent growth conditions and a decrease on metastatic ability (70). In synovial sarcoma, the increased IGF-2 synthesis protects cells from anoikis and is required for tumor formation *in vivo* (71). Another trans-membrane growth factor receptor, the ErbB4 Tyrosine kinase, gets phosphorylated in ES spheroids and its expression is linked to anoikis avoidance, metastatic disease, and bad outcome (72). In RMS, spheroids obtained after cell culture enrichment express stem cell gene markers such as *oct4, pax3, sox2, c-myc*, and *nanog*. It was also found that CD133 was upregulated in these spheres, conferring cells higher resistance to Cisplatin and Chlorambucil *in vivo* (73). In osteosarcoma (OS) cells, anoikis can be induced by zoledronic acid, DNA methylation inhibitors as decitabine or cyclooxygenase-2 inhibitors via PI3K/Akt pathway inhibiting β-catenin, TrkB, and E-cadherin (74–76).

Several of the aforementioned reports present indeed interesting data for a number of plausible targets concerning mitochondrial apoptosis. However, it is worth noting that in most of these cases, apoptotic analyses rely only in AnnexinV (AnnV) tests or caspase-3 activation kits, being uninformative about the precise processes involved. Although extended in the community, when the end-points of AnnV-PI tests are not carefully selected, this could lead to the misidentification of late apoptotic and necrotic cells; similarly, caspase-3 is a common final step in apoptotic cell death that does not imply a single precise activation pathway (Figure 2) (11).

#### The death receptor pathway

Caspase-8 is the most characteristic mediator of the “death receptor pathway” (Figure 2). In this case, the triggers of the apoptotic process are extracellular signals (mostly from the TNF family) and the initiators and mediators encounter not in the mitochondrial outer membrane but rather close to the plasma membrane (77). Besides direct stimulation of cell death, death receptors can also induce specific protein synthesis by means of the NF-κB pathway that balances and even counteracts the apoptotic signaling (78).

TRAIL is a death ligand that has been studied in several sarcomas for therapeutic purposes (79–81). TRAIL-induced apoptosis is regulated by other receptors and downstream effectors including cFLIP and the Bcl-2 family (82–84). The TRAIL receptor, death receptor 5, has been identified as a mediator of chemically induced apoptosis in RMS, synovial sarcoma and leiomyosarcoma, activating several apoptosis triggers (85–87). TNFα and FasL receptors play also a significant role in the survival/apoptotic balance with p21 as critical mediator of the anti-apoptotic effect of TNFα-induced NF-κB (88, 89). Bad, a pro-apoptotic member of the Bcl-2 gene family, has been linked to FasL induced apoptosis in ES (90). Activation of death receptors could be combined with other challenges like doxorubicin, interleukin-12, or immunotoxins (91–93). Some other TNF receptor-related proteins, like NGFR, have been proposed to be crucial in specific sarcomas (94). Thus, there is still a need for a better understanding of the role of the other cell death receptors in sarcomas.

Besides the death receptors themselves, the best strategy to enhance extrinsic apoptosis is repressing NF-κB activation. This rationale has been employed with success against ES and synovial sarcoma (95, 96). Sensitization to apoptosis has also been achieved by re-expressing caspase-8 through demethylation or gene transfer (97).

### Necrosis

Necrosis, in contrast to apoptosis, has been viewed classically as a form of accidental death brought about by injury to the cell by pathogens or toxins. Despite the extended pre-judice, necrosis is more than a mere accidental death (5). Loss of plasma membrane integrity, the “cellular explosion”, is the major morphological feature and characteristic element of necrosis (Figure 1) (9, 98). Non-accidental or “regulated” necrosis has attracted a growing interest in the scientific community in the last years. Necroptosis is the best known phenotype in this group. It is induced by either the activation of death receptors or specific injuries that are followed by the recruitment of the so-called necrosome of which the principal participants are the receptor-interacting protein kinases (RIPK1 and RIPK3), which finally activate the executor MLKL (Figure 2) (99).

Necroptosis is just starting to be studied in sarcomas. It can be distinguished from apoptosis by its distinct morphology and the inability of caspase inhibitors to prevent it (10, 11). In an OS model, RIPK1-mediated necroptosis was confirmed as the main cell death mechanism involved in Shikonin therapy, as only Necrostatin-1 (an inhibitor of RIPK1) was able to induce treatment reversion (100). Basit *et al*. found that Obatoclax (a Bcl-2 inhibitor) treatment in RMS cell lines promoted necroptosis rather than autophagic cell death, being autophagy only a necessary event required for the necrosome assembly (101). So, it becomes clear that there is still a big room for improvement in the accurate characterization of regulated necrosis responses in anti-sarcoma therapy.

### Other scenarios for the cell death drama

The long-standing dichotomy apoptosis-necrosis is in part nothing but a classification artifact. Many times the exact nature of the mechanism triggered relies simply on the intensity of the injury or on the available energy (102). Furthermore, in the cell death landscape, there are other “circumstances” worth of some additional explanation.

A classical example of “double-edged sword” is autophagy, sometimes included as a cell death mechanism, although it usually proceeds as a pro-survival process. Autophagy targets apoptotic-signaling mitochondria for isolation and degradation, thus interrupting the apoptotic outcome. Several proteins cross-link autophagy and apoptosis signaling pathways, being mTOR one of the most studied (103). As a process impacting the energy availability, autophagy also dialogs with necrotic signaling and some reports point to a close relationship with necroptotic triggering (101, 102). Again, it seems to be a question of threshold. In many cases, an excessive autophagy can lead to cell death but this death follows a mixed pattern with parallel apoptotic or necrotic phenotypes. Only when inhibition of autophagy can impede cell death and the final phenotype is considered non-apoptotic cell death, we can classify it as “autophagic cell death” (4, 102). Among the different techniques available, autophagy can be better followed by microscopy assessment of autophagosome formation (11, 104).

To our knowledge, except for some interesting report showing autophagic triggering of necroptosis in RMS (101), no instances of true autophagic cell death have been described in sarcomas yet. Indeed, its role in cancer therapy is still controversial (102). In ES and OS, the protective role of autophagy was insufficient to block apoptotic cell death when triggered by either the intrinsic or the death receptor pathways (105, 106). Autophagy has also been described to be actively removing micronuclei in OS cells, generating an interesting connection with the stabilization of cells recovering from failures during mitosis (107).

Mitotic catastrophe (MC), previously classified as a form of cell death, constitutes a crossroad that could drive cells to die with either apoptotic or necrotic features, go into senescence, or even survive (108). Again, the precise features of the final death phenotype depend on cell context and energy availability (108, 109). The clearest triggers of MC are the dysfunctions of the mitotic spindle. Those dangers are monitored by specific checkpoint proteins determining the final outcome. Thus, cells evading the mitotic arrest have an increase in chromosome instability (110). MC can be easily followed by means of microscopy observation, usually aided with fluorescent markers, video-microscopy, and cell fate imaging analysis.

Proper metaphase arrangement is required for mitosis and is a key process monitored by several checkpoint regulators (Figure 2). BubR1, involved in the mitotic spindle checkpoint, has been shown to be necessary for survival in some RMS cell lines and its knockdown promoted growth suppression and “mitotic catastrophe” but the final outcome was not elucidated (111). Plk1 is another major component of MC signaling: siRNA inhibition of Plk1 killed RMS cells and the chemical inhibitor BI 2536 induced G_2_/M arrest and cell death in OS cell lines (112, 113). Inhibitors of Aurora kinases block the formation of the cleavage furrow, disrupting cytokinesis, and killing leiomyosarcoma and synovial sarcoma cells (114, 115). Chk1 blockade with CEP-3891 caused an abrogation of the S and G_2_ checkpoints after ionizing radiation, giving rise to nuclear fragmentation as a consequence of defective chromosome segregation and promoting cell death (116). Many active drugs tested in sarcoma cells have been described to disrupt normal cell cycle. Those compounds range from small molecules or plant derivatives, to cell cycle kinase inhibitors, viral proteins etc. Several studies showed cell cycle arrest and changes in the levels of MC mediators as Survivin. For example, Keyomarsi’s group showed that combined therapy with doxorubicin and roscovitine in synovial sarcoma and leiomyosarcoma induced a synergistic increase in autophagy in addition to a marked arrest in G_2_/M (117). Links between MC and autophagy have also been commented previously for OS (107). In any case, it would be desirable to perform an exhaustive mitotic study or cell fate analysis together with the proper assessment of the nature of cell cycle blockade (metaphase arrest, G_2_ stop, or even senescence).

## Cell Death Mechanisms in Anti-Sarcoma Clinical Trials

New targeted therapies linked to key cell death mechanisms are continuously being developed (118). Preferred to cytostatic alternatives, cell death induction is the goal of the vast majority of cancer treatments. And among the known mechanisms, apoptosis is the center of therapeutic developments (118). As a non-inflammatory mechanism, apoptosis is traditionally considered cleaner than necrosis, but its exact relevance in overall therapeutic success is uncertain. Necrosis, due to its pro-inflammatory nature, has been regarded as a back door for metastatic cells to escape from the primary tumor (3, 119). But, depending on the circumstances, necrosis could be effective enough to induce tumor clearance (120). Conversely, a particular apoptotic phenotype with the ability to trigger immune response against cancer cells has been described (119). Moreover, classic chemotherapeutic agents are shown to induce apoptosis by interfering with the normal cell division processes and this could lead to the triggering of MC (108, 109, 121). Induction of MC *vs*. direct apoptosis triggering depends of the effective drug concentration within the cells and thus, could be different among the tumor mass (122). MC drives most of the cells to major death mechanisms but opens the gates for the appearance of new stable karyotypes translating into perhaps new resistant cancer clones (108, 123, 124).

The treatment of advanced sarcomas is based on classic chemotherapeutic agents: anthracyclines and ifosfamide as first option and, after progression, other agents like gemcitabine in combination with docetaxel (or Dacarbazine) and trabectedin. The benefit of chemotherapy is well-known, but limited, because a high percentage of patients die due to the disease in approximately 1 year from diagnosis (125, 126).

In the past years, several sarcoma-focused clinical trials have evaluated the activity in monotherapy of novel drugs with known connections to a particular cell death mechanism (Table 1). So far, only two phase III trials have been reported, reflecting that targeted therapies have been mostly developed in recent years and remain in a pre-clinical stage (127–137). The first trial was focused on the mTOR signaling pathway, which links apoptosis with autophagy (102, 103). The study evaluated the role of ridaforolimus as maintenance therapy after clinical benefit to chemotherapy (133). The other trial analyzed the activity of Pazopanib (a multitargeted kinase inhibitor) in pre-treated soft-tissue sarcoma patients (136).

**Table 1:** **Summary of already published clinical trials that evaluate target therapies in sarcomas, classified regarding the mechanism of action**.

	Mechanism of action	Drugs	Trial (reference)	Study population	Benefits	Common severetoxicities
**Apoptosis**	**PARP inhibitors**	Olaparib	Phase II (127)	Recurrent/metastatic adult ES (failure to prior CH), *n* = 12 patients	NO responses SD: 4 patients, TTP: 5.7 weeks	No significant toxicities
	**Heat shock protein inhibitors**	Retaspimycin (Hsp-90 INH)	Phase I (128)	Metastatic and/or unresectable STS, *n* = 54 patients	PR: 2 patients (proof of clinical activity)	Grade 3–4: Fatigue Nausea and vomiting Headache Artharalgia
	**Proteaseome inhibitor**	Bortezomib	Phase II (129)	Metastatic OS, ES, RMS, and STS with no prior treatment for advanced disease, *n* = 25 patients	Lack of benefit (trial prematurely closed)	Grade 3–4: Neuropathy Asthenia Myalgias
	**MDM2 inhibitor**	RG7112	Proof of mechanism study (130)	WDLS or DDLS with MDM2 amplification receive RG7112 prior to surgery, *n* = 20 patients	SD: 14 patients, IHQ: activation of p53 pathway	Grade 3–4 Neutropenia Thrombocytopenia
			Phase I (131)	Phase I trial with extension cohort for sarcoma patients, *n* = 30 (sarcoma patients)	Metabolic responses (PET-CT) IHQ: activation of p53 (MDM2-independent)	Grade 3–4 Cytopenias
	**PI3K-AKT-mTOR pathway inhibitors**	Ridaforolimus (mTOR INH)	Phase II (132)	Pre-treated advanced bone and STS, *n* = 212 patients	RR: 1.9%, clinical benefit: 28.8%	Grade 3–4 Fatigue Stomatitis Hypertriglyceridemia Anemia Thrombocytopenia
			Phase III (133)	Advanced bone and STS with clinical benefit to previous CH were randomized to maintenance Ridaforolimus or Placebo, *n* = 711 patients	Improvement in PFS (17.7 weeks with Ridaforolimus vs. 14.6 weeks with Placebo, HR: 0.72, *p*: 0.001)	Similar to previous study
		Everolimus (mTOR INH)	Phase II (134)	Pre-treated advanced bone and STS, *n* = 41 patients	Poor clinical activity	Grade 3–4 Hyperglicemia Stomatitis Pain Asthenia
	**Anti-angiogenic therapy**	Sorafenib (VEGFR2, VEGFR3, PDGFR, and c-Kit INH)	Phase II (135)	Pre-treated advanced STS, *n* = 101 patients	RR: 14.5%, SD: 32.9% (leiomyosarcoma better PFS)	Grade 3–4 Fatigue Diarrhea Hand–foot Syndrome Nausea and vomiting
		Pazopanib (VEGFR-1, VEGFR-2, VEGFR-3, PDGFR, and c-Kit INH)	Phase III (136)	Pre-treated non-adipocytic STS randomized to PAZOPANIB vs. PLACEBO, *n* = 369 patients	Improvement in PFS (4.6 months with PAZOPANIB vs. 1.6 months with Placebo, HR: 0.31, *p* < 0.0001)	Grade 3–4 Asthenia Hypertension Anorexia Alteration of transaminases

**Mitotic catastrophe**	**CDK inhibitors**	Palbociclib (CDK4 and CDK6 INH)	Phase II (137)	WDLS or DDLS with CDK4 amplification and pRb expression	66% of patients free of PD at 12 weeks	Grade 3–4 Anemia Neutropenia Thrombocytopenia

*CH: chemotherapy, DDLS: dedifferentiated liposarcoma, HR: hazard ratio, INH: inhibitor, MPNST: malignant peripheral nerve sheath tumor, PFS: progression-free survival, RR: response rate, SD: stabilization disease, STS: soft-tissues sarcoma, TTP: time to progression, WDLS: well-differentiated liposarcoma*.

It is easily noticeable that many of the targets mentioned above have still not reached the clinical trial stage in sarcomas. Further research should be aimed to fill that gap by a better description of the pre-clinical effects in terms of quantity and quality (type, characterization, assessment of resistant phenotypes, etc.) of the induced cell death. A summary of the ongoing clinical trials in sarcomas are included in Table 2.

**Table 2:** **Summary of clinical trials that are ongoing and evaluate target therapies in sarcomas, classified regarding the mechanism of action**.

		Ongoing trials specific for sarcomas	Status www.clinicaltrials.gov	Identifier www.clinicaltrials.gov
**Apoptosis**	**PARP inhibitors**	ESP1/SARC025 global collaboration: a Phase I study of a combination of the PARP inhibitor, niraparib, and temozolomide in patients with previously treated, incurable Ewing sarcoma	Ongoing, but not recruiting	NCT02044120
		Olaparib in adults with recurrent/metastatic Ewing’s sarcoma.	Ongoing, but not recruiting.	NCT01583543
	**Heat shock protein inhibitor**	A trial of ganetespib Plus sirolimus: phase 1 includes multiple sarcoma subtypes and Phase 2 MPNST	Ongoing, but not recruiting	NCT02008877
	**PI3K-AKT-mTOR pathway inhibitors**	Phase II study of everolimus in children and adolescents with refractory or relapsed osteosarcoma	Recruiting	NCT01216826
		Phase II open label, non-randomized study of Sorafenib and everolimus in relapsed and non-resectable osteosarcoma (SERIO)	Ongoing, but not recruiting	NCT01804374
		Study of everolimus with bevacizumab to treat refractory malignant peripheral nerve sheath tumors	Ongoing, but not recruiting	NCT01661283
		Phase II study of everolimus in children and adolescents with refractory or relapsed rhabdomyosarcoma and other soft tissue sarcomas	Recruiting	NCT01216839
	**Anti-angiogenic therapy**	Sorafenib tosylate, combination chemotherapy, radiation therapy, and surgery in treating patients with high-risk stage IIB–IV soft tissue sarcoma	Recruiting	NCT02050919
		Pazopanib hydrochloride followed by chemotherapy and surgery in treating patients with soft tissue sarcoma	Recruiting	NCT01446809
		Activity and tolerability of pazopanib in advanced and/or metastatic liposarcoma. a phase ii clinical trial	Recruiting	NCT01692496
		Study of pazopanib in the treatment of osteosarcoma metastatic to the lung	Recruiting	NCT01759303
		Study of pre-operative therapy with pazopanib (votrient^®^) to treat high-risk soft tissue sarcoma (NOPASS)	Recruiting	NCT01543802

**Mitotic catastrophe**	**Aurora-kinase inhibitors**	Alisertib in treating patients with advanced or metastatic sarcoma	Recruiting	NCT01653028
	**CDK inhibitors**	PD0332991 in patients with advanced or metastatic liposarcoma	Recruiting	NCT01209598

## Concluding Remarks

As often happens with research on rare diseases, sarcoma research suffers from funding shortage and delayed implementation of technical advances. But there is also an urgent need to improve current therapeutic modalities in sarcomas and reduce their burden. Additionally, due to their heterogeneity, sarcoma models are very difficult to compare among them. Those constrains define sarcoma research today. Cell death induction is the basis of cancer therapy, but we are still far from understanding the mechanisms of cell death signaling in sarcomas. The relatively low attention paid to particular phenomena like autophagy or MC, with crucial roles in therapy success, is symptomatic that we need to get back to the laboratory benches and improve our methods (3, 118, 124). We abuse too often of indirect tests, easy to read-out in flow cytometers, or high-content analyzers. And perhaps, we rely too much in bibliographic data, not looking for the actual connections between our treatments and the specific cell death trigger.

Sarcoma research needs the implementation of a better determination of cell death mechanisms. The definition of the nature of cell death is not a vain effort as the differences in mechanisms could have tremendous consequences in terms of chemo-resistance or in immunogenic potential (108, 119, 123, 124). We need to dedicate more time to define cell death circumstances, but sometimes it seems that this attention only happens when researchers are faced with unusual/specific cell death signals (death receptors, MC, necroptosis etc.) while relying in the bulk caspase-3 or AnnV-PI kits for the rest of the occasions.

The extra work we are proposing is neither difficult nor exhausting, as it requires only to spend a little time looking “what” actually happens to our cells (and “when”). Cell death is evident to the trained eye by merely observing the cells in the cell culture room’s inverted microscope (Figure 1). Then, there are enough valuable tests, clear and easy to perform, for the major cell death pathways (138). Performed in the correct set of end-points a simple DAPI staining would serve to determine whether we are facing apoptosis, necrosis, or MC (10, 11). Therefore, we encourage researchers to perform those tests and include their results in their publications prior to embark themselves into more complex analysis about the intimacy of cell physiology. Let’s concentrate on describing better “what” is happening before moving on solving “how” it is happening.

## Conflict of Interest Statement

The authors declare that the research was conducted in the absence of any commercial or financial relationships that could be construed as a potential conflict of interest.

## Abbreviations

AIF, apoptosis inducing factor; Akt, protein kinase B; Apaf-1, apoptotic protease activating factor 1; Bak, Bcl-2 homologous antagonist/killer; Bax, Bcl-2-associated X protein; Bcl-2, B-cell lymphoma 2; Bcl-x_L_, B-cell lymphoma-extra large; BubR1, mitotic checkpoint serine/threonine-protein kinase BUB1 beta protein; CD133, prominin-1; CD99, cluster of differentiation 99 protein; cFLIP, cellular FLICE inhibitory protein; Chk1, checkpoint kinase 1; c-Myc, cellular avian myelocytomatosis viral oncogene homolog; DAPI, 4′,6-diamidino-2-phenylindole; DRAL, downregulated in rhabdomyosarcoma LIM protein; ERG, protein encoded by erythroblast transformation-specific related gene; ERK, extracellular signal-regulated protein kinase; EWS, Ewing sarcoma RNA-binding protein; FasL, fas ligand protein; FLI1, Friend leukemia integration 1 transcription factor; FOXO1, forkhead box protein O1; HDAC1, histone deacetylase 1; IGF-1R, insulin-like growth factor 1 receptor; IHQ, immunohistochemistry; JAK, Janus kinase; MC, mitotic catastrophe; MDM2, mouse double minute 2 homolog protein; MEK, mitogen-activated protein kinase kinase; miRNA, micro RNA; MLKL, mixed lineage kinase domain-like protein; mTOR, mammalian target of rapamycin protein; NF-κB, nuclear factor kappa-light-chain-enhancer of activated B cells protein; NGFR, low-affinity nerve growth factor receptor; Noxa, Phorbol-12-myristate-13-acetate-induced protein 1; *oct4*, octamer-binding transcription factor 4; p21, cyclin-dependent kinase inhibitor 1; PARP, poly (ADP-ribose) polymerase; PAX, protein encoded by paired box gene; PD, progression disease; PDGFR, platelet-derived growth factor receptor; PET-CT, positron emission tomography; PI, propidium iodide; PI3K, phosphatidylinositol-4,5-bisphosphate 3-kinase; Plk1, polo-like kinase 1; PM, plasma membrane; PR, partial response; pRb, retinoblastoma protein; puma, p53 upregulated modulator of apoptosis; RAF, *raf* proto-oncogene serine/threonine-protein kinase; RANK, receptor activator of nuclear factor κB; ROS, reactive oxygen species; *sox2*, sex determining region Y-box 2; SSX, protein encoded by synovial sarcoma X breakpoint gene; STS, soft tissue sarcoma; SYT, protein encoded by synovial sarcoma translocation on chromosome 18 gene; TNFR, tumor necrosis factor receptor; TRAIL, TNF-related apoptosis-inducing ligand; VEGFR-2, vascular endothelial growth factor receptor 2.
